# Multi-Omics Analysis of the Therapeutic Value of MAL2 Based on Data Mining in Human Cancers

**DOI:** 10.3389/fcell.2021.736649

**Published:** 2022-01-17

**Authors:** Jing Yuan, Xiaoyan Jiang, Hua Lan, Xiaoyu Zhang, Tianyi Ding, Fan Yang, Da Zeng, Jiahui Yong, Beibei Niu, Songshu Xiao

**Affiliations:** ^1^ Department of Gynecology and Obstetrics, Third Xiangya Hospital, Central South University, Changsha, China; ^2^ School of Life Science and Technology, Institute for Regenerative Medicine, Shanghai East Hospital, Tongji University, Shanghai, China; ^3^ Scientific Research Center, Xinhua Hospital, Affiliated to Shanghai Jiao Tong University School of Medicine, Shanghai, China

**Keywords:** ovarian cancer, T-cell differentiation protein 2 (MAL2), CRISPR/Cas9, epithelial-mesenchymal transition (EMT), proliferation, multi-omics

## Abstract

Recent studies have reported that T-cell differentiation protein 2 (MAL2) is an important regulator in cancers. Here, we downloaded data from multiple databases to analyze MAL2 expression and function in pan-cancers, especially in ovarian cancer (OC). Gene Expression Profiling Interactive Analysis (GEPIA) databases was used to examine MAL2 expression in 13 types of cancer. Kaplan–Meier plotter database was used to analyze the overall survival rate of MAL2 in pan-cancers. The Catalog of Somatic Mutations in Cancer (COSMIC), cBioPortal, and UCSC databases were used to examine MAL2 mutation in human cancers. Metascape, STRING, and GeneMANIA websites were used to explore MAL2 function in OC. Furthermore, ggplot2 package and ROC package were performed to analyze hub gene expression and undertake receiver operating characteristic (ROC) analysis. Drug sensitivity of MAL2 in OC was examined by the GSCALite database. In order to verify the results from databases above, real-time quantitative polymerase chain reaction (qRT-PCR) and western blotting were conducted to detect the expression of MAL2 in OC cells. CRISPR/Cas9 system was used to knockout the MAL2 gene in the OC cell lines HO8910 and OVCAR3, using specific guide RNA targeting the exons of MAL2. Then, we performed proliferation, colony formation, migration, and invasion assays to investigate the impact of MAL2 in OC cell lines *in vivo* and *in vitro*. Epithelial-mesenchymal transition (EMT)-associated biomarkers were significantly altered *in vitro* via western blotting and qRT-PCR. Taken together, we observed that MAL2 was remarkably dysregulated in multiple cancers and was related to patient overall survival (OS), mutation, and drug sensitivity. Furthermore, experimental results showed that MAL2 deletion negatively regulated the proliferation, migration, invasion, and EMT of OC, indicating that MAL2 is a novel oncogene that can activate EMT, significantly promote both the proliferation and migration of OC *in vitro* and *in vivo*, and provide new clues for treatment strategies.

## Introduction

The T-cell differentiation protein 2 (MAL2) gene, located on human chromosome 8q24, is a 19-kDa membrane protein with four transmembrane domains belonging to the MAL protein family (Fanayan et al., 2009) that participates in transporting apical vesicles. Through yeast two-hybrid expression screening of a human breast cancer library, MAL2 was identified as the molecular chaperone of tumor protein D52-like protein (TPD52) and MUC1 (Fanayan et al., 2009; Li et al., 2017). MAL2 protein is expressed in a variety of epithelial cells, peripheral neurons, mast cells, and dendritic cells and is crucial in immunity, tumor development, and other physiological and pathological processes (Marazuela et al., 2004). The latest studies revealed that a high expression of MAL2 can be detected in breast cancer tissues and cells (Bhandari et al., 2018) and that MAL2 participates in regulating cell proliferation, invasion, and metastasis; promotes the malignant progression of cancer; and is significantly associated with clinical pathology or prognosis. The prognosis of patients with high expression of MAL2 is worse than that of patients with low expression (Byrne et al., 2010; In et al., 2014; Liu et al., 2017; Wilson et al., 2001). MAL2 has been reported to regulate liver polarization (López-Coral et al., 2020), which plays a role in regulating cell protein transport, which alters protein distribution and ultimately affects cell morphology, signaling pathways, and cell migration. Therefore, MAL2 may be a potential therapeutic target and prognostic factor for breast cancer. However, there was no systematic research analyzing the role of MAL2 in human cancers.

Tumors gradually become a leading cause of death all over the world. In our present research, we observed a strong association between MAL2 and the survival rate and gene mutation rate in patients of ovarian cancer (OC). According to the latest statistics, OC ranks third among malignant tumors of the female reproductive system in terms of incidence rate (Siegel et al., 2021). Most OC cases are characterized by dormant onset and rapid progression. Most patients show non-specific symptoms or no symptoms at all when cancer cells have invaded other organs of the body, which makes it difficult to detect during the early stages. Moreover, there is no effective screening method for the early detection of OC; thus, almost 80% of OC cases are diagnosed at an advanced stage (Bristow and Chi, 2006; Cunnea et al., 2017; Smith et al., 2018). At present, the standard treatment for OC is mainly surgery supplemented with platinum-based chemotherapy (Luvero et al., 2014). However, almost ∼70% of tumors have spread to distant tissues, such as pelvic and abdominal organs (Feng et al., 2019), at the time of diagnosis, resulting in a poor prognosis. The 5-year survival rate of OC patients is only approximately 40% (Armbruster et al., 2018). Therefore, it is urgent to decipher the intricate pathogenesis of OC to shed light on potential novel therapeutic targets and find reliable biomarkers or therapeutic targets.

In our study, we first analyzed the MAL2 expression and clinical value in pan-cancers using Gene Expression Profiling Interactive Analysis (GEPIA) and Kaplan–Meier plotter databases. MAL2 gene mutation, methylation, function enrichment, protein–protein interaction (PPI), gene–gene interaction (GGI), cancer pathway activation, and drug sensitivity were comprehensively evaluated. From the systematic analysis of MAL2 in pan-cancers, we found that MAL2 was significantly overexpressed and present with high mutation rate in OC samples, indicating that MAL2 may play a role in OC. We next examined the function of MAL2 in OC by performing cell experiments. Results suggested that MAL2 was upregulated in OC cell lines and has the ability to promote cell proliferation, migration, and invasion and regulate epithelial-mesenchymal transition (EMT)-related proteins. In the present study, we elucidated the function of MAL2 in OC occurrence and development.

## Materials and Methods

### Expression Analysis and Clinical Correlation Analysis

GEPIA (http://gepia.cancer-pku.cn/index.html) is an online tool providing gene expression data based on The Cancer Genome Atlas (TCGA) and Genotype-Tissue Expression (GTEx) projects. Kaplan–Meier plotter (www.kmplot.com) collected gene expression data and clinical data from multiple databases like GEO, EGA, TCGA, and other public databases. It provides a server for survival rate analysis.

### Mutation and Methylation Analysis

The Catalog of Somatic Mutations in Cancer (COSMIC) database (https://cancer.sanger.ac.uk/cosmic/) provides data about coding mutations, non-coding mutations, genome rearrangements, fusion genes, etc. in human genome. cBioPortal for cancer genomics (http://www.cbioportal.org/) is an online website collecting multiple kinds of mutation data of human cancers. In this study, two databases were used to examine MAL2 mutations in human cancers. The UCSC Xena tool (xena.ucsc.edu) was used to analyze the correlation between Illumina’s human methylation and gene expression in OC patients.

### Function Enrichment and PPI and GGI Network Construction

Metascape (www.genemania.org) is a powerful tool to perform Gene Ontology (GO) and Kyoto Encyclopedia of Genes and Genomes (KEGG) enrichment analysis between MAL2 and neighboring genes. STRING (www.string-db.org) is a prediction server to analyze PPI to provide insights into the mechanisms of MAL2 to OC. GeneMANIA (www.genemania.org) is an online analysis server for obtaining a list of related genes with associated functions to MAL2 from constructing an interaction network to clarify the relationship between MAL2 and their interacting genes.

### R Studio

The expression analysis comparing transcript level in cancer tissues with matched normal tissues was accomplished using the ggplot2 package of R3.6.3 version. Data was downloaded from TCGA and GTEx in transcripts per million (TPM) format, and receiver operating characteristic (ROC) analysis of 10 MAL2-related hub genes and calculating the areas under the curve (AUC) were accomplished using the ROC package of R3.6.3 version.

### Analysis of Cancer Pathway and Drug Sensitivity

GSCALite (http://bioinfo.life.hust.edu.cn/web/GSCALite/) is a powerful online website providing data about cancer gene expression, methylation, single-nucleotide variation, cancer pathway activity, and drug sensitivity. In this study, the GSCALite website was used to analyze the influence of MAL2 and its neighboring genes in activating cancer pathway and conducting drug sensitivity.

### Cell Culture and Transfection

The human OC cell lines HO8910 and OVCAR3 and the human ovarian epithelial cell lines IOSE80 and 293T were purchased from the American Type Tissue Culture Collection (ATCC) (Manassas, VA, United States). HO8910, OVCAR3, and IOSE80 cells were cultured in RPMI 1640 medium (Invitrogen, New York, United States, Cat No. 27016021) supplemented with 10% certified heat-inactivated fetal bovine serum (FBS; Gibco, New York, United States, Cat No. 10100147) and 100 U/ml penicillin/streptomycin (Gibco, New York, United States, Cat No. 10378016) and incubated at 37°C in a humidified incubator containing 5% CO_2_ (Thermo Fisher Scientific, Kalamazoo, MI). The 293T cells were cultured in DMEM (Invitrogen, New York, United States, Cat No. 11965092).

### CRISPR/Cas9 Protocol and Lentivirus Transfection

The single guide RNA sequences were designed according to the exon1 of human MAL2 gene and predicted by the online tool created by Prof. Zhang (http://crispr.mit.edu/). The sgRNA sequences were sgRNA-1 GAA​GGA​CAC​GGC​GGG​GTT​CGG​GG and sgRNA2 CTC​GGG​CGC​CTT​CGT​CTG​CCT​GG. The sgRNAs were inserted into the LentiCRISPR v2 vector to construct hu6-sgRNA-EF1A-hspCas9-Flag-puro-WPRE plasmids. Targeting plasmids and packaging plasmids were cotransfected into 293T cells using Lipofectamine 3000 (Invitrogen, New York, United States, Cat No. L3000015). After purification and ultracentrifugation, the supernatant was collected for cell infection. Then, 5 μg/ml puromycin was added 72 h after infection, and the surviving cells were collected for positive clones selecting.

### RNA Extraction and Real-Time Quantitative Polymerase Chain Reaction

Total RNA was isolated using TRIzol reagent (Sigma, Cat No. T9424) and then reverse transcribed into cDNA using the Revert Aid First Strand cDNA Synthesis Kit (Thermo Scientific, Massachusetts, United States, Cat No. 18064022). Real-time PCR was performed using qPCR SYBR Green Master Mix (Yeasen, Shanghai, China, Cat No. 11201ES08) and analyzed with a LightCycler 96 quantitative PCR system (Roche). The relative mRNA expression level of genes was calculated using the 2^−ΔΔCt^ method after normalization to GAPDH, which served as an internal loading control.

The primer sequences used were as follows:

**Table:** 

Gene	Forward primer	Reverse primer
MAL2	TTC​TGT​TCG​GGG​GTC​TTG​TC	CAC​CAT​GCC​AGA​GAG​GAA​CA
GAPDH	TGA​CTT​CAA​CAG​CGA​CAC​CCA	CAC​CCT​GTT​GCT​GTA​GCC​AAA
CDH1	GCT​GGA​CCG​AGA​GAG​TTT​CC	CAA​AAT​CCA​AGC​CCG​TGG​TG
CDH2	AGA​ACG​CCA​GGC​CAA​ACA​AC	ATT​CGT​CGG​ATT​CCC​ACA​GG
VIM	CTG​GAT​TCA​CTC​CCT​CTG​GTT	TCG​TGA​GCT​GAG​AAG​TTT​CG

### Western Blotting

Total protein was lysed on ice with RIPA lysis buffer (Yeasen, Shanghai, China, Cat No. 20101ES60) supplemented with 1% PMSF (Yeasen, Shanghai, China, Cat No. 20104ES03) for 30 min to prepare the cell suspension; then, the sample was centrifuged at 12,000 rpm for 15 min at 4°C, and the protein concentration was quantified with a BCA kit (Yeasen, Shanghai, China, Cat No. 20201ES76). Proteins were separated by 8% or 12% SDS-PAGE and transblotted to PVDF membranes (Millipore, Billerica, United States, Cat No. HVLP04700). After being blocked in 5% non-fat milk for 1.5 h at room temperature, the membranes were incubated with primary antibodies at 4°C with gentle shaking overnight. The target proteins were detected by using specific antibodies: rabbit anti-human MAL2 polyclonal antibody (Abcam, Shanghai, China, 1:250 dilution, Cat No. ab75347), rabbit anti-human N-cadherin polyclonal antibody (HuaBio, Zhejiang, China, 1:2,500 dilution, Cat No. ER0503), mouse anti-human vimentin polyclonal antibody (HuaBio, Zhejiang, China, 1:2,500 dilution, Cat No. M1412-1), and rabbit anti-human E-cadherin polyclonal antibody (HuaBio, Zhejiang, China, 1:2,500 dilution, Cat No. 0407-25). Rabbit anti-human GAPDH polyclonal antibody (Yeasen, Shanghai, China, 1:100,000 dilution, Cat No. 30202ES40) and rabbit anti-human β-tubulin polyclonal antibody (Yeasen, Shanghai, China, 1:100,000 dilution, Cat No. 30302ES20) were chosen as internal controls. After the membranes were washed three times for 20 min each time, secondary antibodies were used: goat anti-rabbit immunoglobulin G (Yeasen, Shanghai, China, 1:100,000 dilution, Cat No. 33107ES60) and goat anti-mouse immunoglobulin G (Yeasen, Shanghai, China, 1:100,000 dilution, Cat No. 33207ES60). Secondary antibodies were added to the membranes to incubate at room temperature for 2 h. The blots were visualized with ECL reagent (Yeasen, Shanghai, China, Cat No. 36208ES60) according to the manufacturer’s protocol.

### Cell Counting Kit-8 Assay

The cells were transferred into 96-well plates (100 μl cell suspension per well) at a density of 1,000 cells/well with triplicate wells for each cell line and incubated into a humidified incubator (Thermo Fisher). After 3 h, 10 μl of Cell Counting Kit-8 (CCK-8) reagent (Yeasen, Shanghai, China, Cat No. 40203ES76) was added to each well, and the cells were incubated for two more hours. Then, an iD3 microplate reader was used to measure the absorbance [optical density (OD) value] at 450 nm. Thereafter, the above steps were repeated, and the OD value was measured at 0, 24, 48, 72, and 96 h.

### Colony Formation Assay

The cells were counted and adjusted to 1,000 cells/well in six-well plates and then placed into a humidified incubator. Two replicate wells were set for each group, and the cells were cultured for 10–14 days. After the cells formed obvious colonies, the cells were washed with phosphate-buffered saline (PBS), fixed in 70% methanol, stained with crystal violet, and photographed. Finally, GraphPad was used to analyze the number of cell colonies in each well.

### Cell Apoptosis Assay

Cells were seeded at a density of 1 × 10^5^ cells/well in a 12-well plate. At the 24-h point, cell morphology was assessed using the Apoptosis and Necrosis Assay Kit (Beyotime, Shanghai, China, Cat No. C0003). The cells were stained with Hoechst 33342 and propidium iodide (PI) according to the manufacturer’s protocols and then visualized by fluorescence microscopy. An Annexin-V-FITC/PI Cell Apoptosis Detection Kit (Meilunbio, Dalian, China, Cat No. MA0220-2) was used to detect the cells’ apoptotic rate according to the manufacturer’s direction. Afterward, the apoptotic rate of cells was measured within 1 h by a flow cytometer (BD Biosciences).

### Transwell Assay

Transwell invasion chambers with 8-µm pores (Corning, New York, United States, Cat No. 3422) were precoated with diluted Matrigel (3:100, BD Biosciences, United States, Cat No. 356234) and incubated at 37°C for over 1 h. A total of 8 × 10^4^ cells suspended in 200 μl medium per well without serum was added to the upper chamber of 24-well plates, and the lower chamber was filled with 600 μl complete RPMI-1640 or DMEM containing 10% FBS. The chambers were incubated at 37°C for 48 h. Then, the cells on the surface of the membrane were counted and imaged by microscopy (Olympus, Tokyo, Japan) after fixation with 70% methanol and staining with crystal violet.

### Wound Healing Assay

The cells were plated in six-well plates and incubated until they formed a 100% confluent cell monolayer. Then, the cells were scratched with sterile 10-μl pipette tips and washed twice gently with PBS. A serum-free medium was applied to culture cells for 48 h. The wound areas were captured by microscopy (Olympus, Tokyo, Japan) at 0, 24, and 48 h.

### Tumor Growth in the OVCAR3 Ovarian Cancer Xenograft Model

Female BALB/c nude mice aged 4 weeks were obtained from the Shanghai Experimental Animal Center, Chinese Academy of Sciences. All animals were raised in a sterile environment and randomly divided into three groups (*n* = 5). Then, 5 × 10^6^ OVCAR3 cells were suspended in 200 μl PBS and injected subcutaneously into the nude mice. Three weeks after injection, the mice were sacrificed by cervical dislocation, and the size and quality of tumor tissue *in vivo* were accurately measured.

### Statistical Analysis

Data were represented as mean ± SD using SPSS 19.0 (SPSS, Inc., Chicago, IL, United States). *P* values <0.05 (∗) and *p* < 0.01 (∗∗) were considered statistically significant. The differences among groups were statistically evaluated by Student’s *t* test or one-way analysis of variance (ANOVA). A two-tailed *p* < 0.05 was considered to indicate significance in all tests.

## Results

### Pan-Cancer Analysis of MAL2 Expression and the Prognostic Value

**FIGURE 1:**
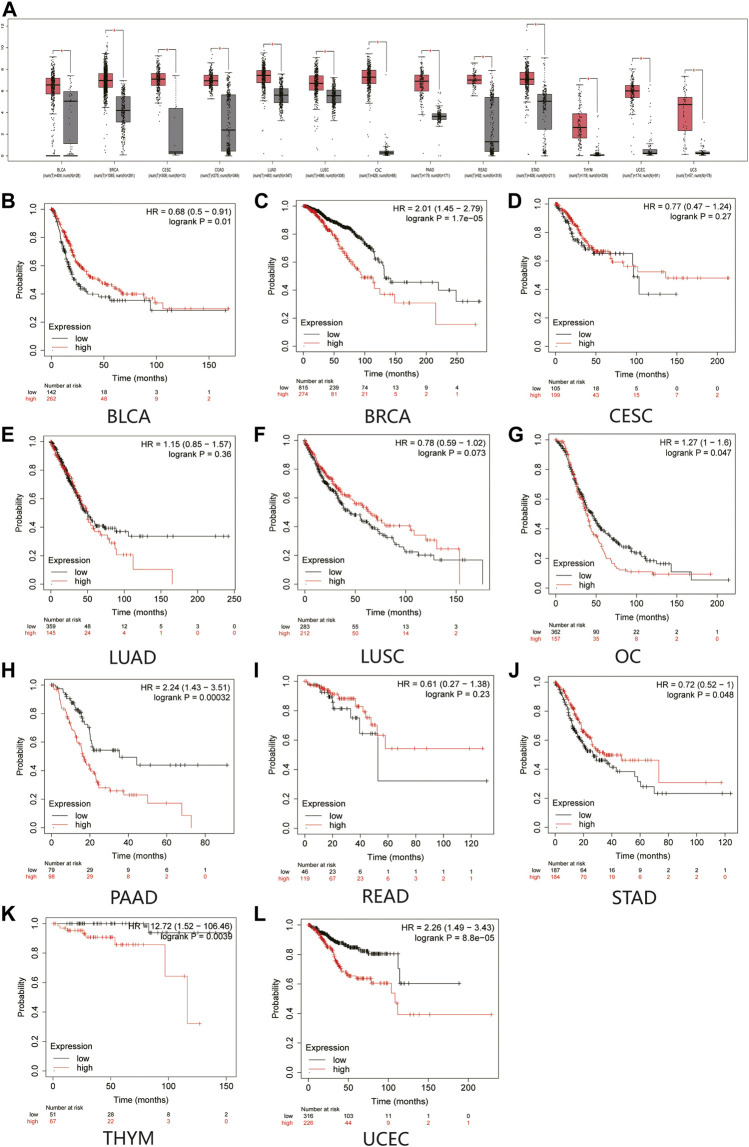
Expression analysis and the overall survival (OS) analysis for MAL2 in multiple cancer. **(A)** MAL2 expression in 13 types of human cancer from GEPIA dataset. **(B–L)** Kaplan–Meier analysis of the overall survival of MAL2 in BLCA **(B)**, BRCA **(C)**, CESC **(D)**, LUAD **(E)**, LUSC **(F)**, OC **(G)**, PAAD **(H)**, READ **(I)**, STAD **(J)**, THYM **(K)**, and UCEC **(L)**. *p < 0.05. MAL2, T-cell differentiation protein 2; GEPIA, Gene Expression Profiling Interactive Analysis; BLCA, bladder urothelial carcinoma; BRCA, invasive breast carcinoma; CESC, cervical squamous cell carcinoma; LUAD, lung adenocarcinoma; LUSC, lung squamous cell carcinoma; OC, ovarian cancer; PAAD, pancreatic adenocarcinoma; READ, rectal adenocarcinoma; STAD, stomach adenocarcinoma; THYM, thymoma (THYM); UCEC, uterine corpus endometrial carcinoma.

First, we confirmed the expression of MAL2 in human cancer tissues by comparing the transcriptome data between pan-cancer tissues and paracancerous tissues from TCGA using GEPIA database. The data showed that MAL2 is highly expressed in pan-cancers, including bladder urothelial carcinoma (BLCA), invasive breast carcinoma (BRCA), cervical squamous cell carcinoma (CESC), lung adenocarcinoma (LUAD), lung squamous cell carcinoma (LUSC), OC, pancreatic adenocarcinoma (PAAD), rectal adenocarcinoma (READ), stomach adenocarcinoma (STAD), thymoma (THYM), and uterine corpus endometrial carcinoma (UCEC), and was remarkably upregulated in two cancer types, involving OC and UCEC (Figure 1A). Taken together, MAL2 was overexpressed in different types of cancer, indicating that MAL2 may be a potential crucial regulator of carcinogenesis in cancer types mentioned above.

Next, Kaplan–Meier plotter database was used to detect the prognostic value of MAL2 in pan-cancer (Figure 1B–L). Survival analysis revealed that a high expression of MAL2 was correlated with worse overall survival rate of BRCA (*p* = 1.7e−05), OC (*p* = 0.047), PAAD (*p* = 0.00032), THYM (*p* = 0.0039), and UCEC (*p* = 8.8e−05), while CESC (*p* = 0.27), LUAD (*p* = 0.36), LUSC (*p* = 0.073), and READ (*p* = 0.23) showed no statistical significance of MAL2 for predicting prognosis of patients. Taken together, MAL2 may be utilized as a potential therapeutic target and unfavorable prognostic biomarker in patients with OC.

### MAL2 Mutation in Human Cancers

**FIGURE 2:**
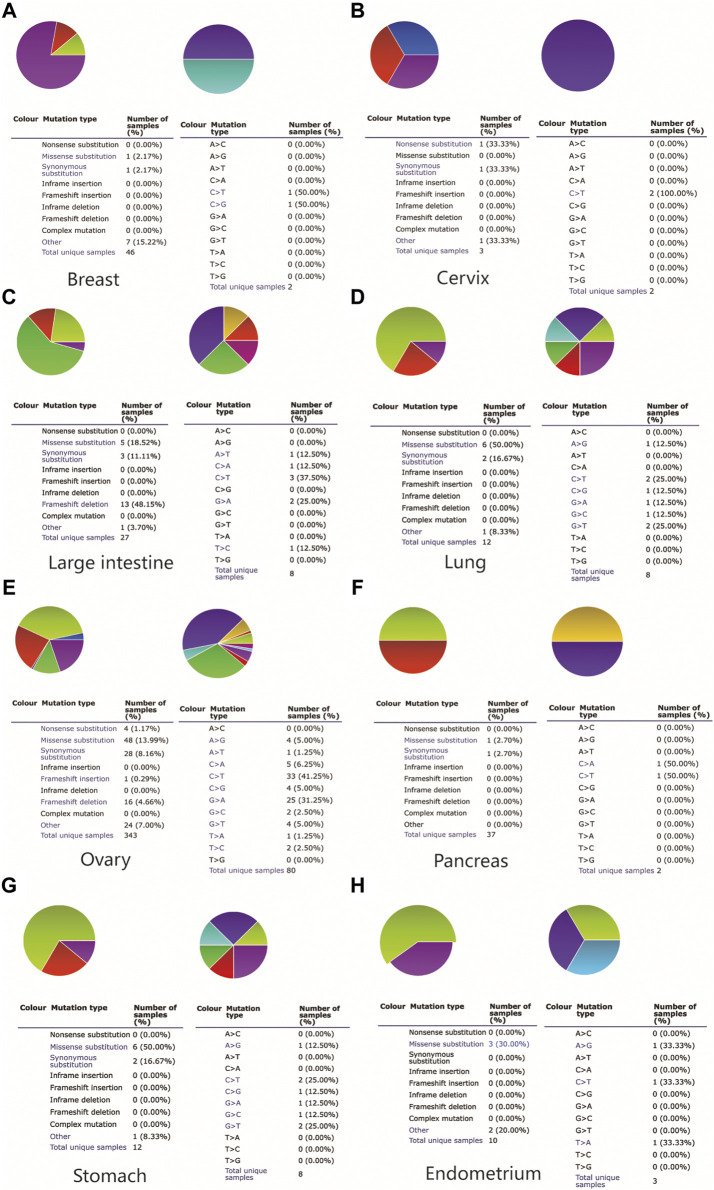
The somatic mutation of MAL2 in pan-cancers. A pie chart showing the percentage of the different mutation types of MAL2 in the breast **(A)**, cervix **(B)**, large intestine **(C)**, lung **(D)**, ovary **(E)**, pancreas **(F)**, stomach **(G)**, and endometrium **(H)** according to the Catalog of Somatic Mutations in Cancer (COSMIC) database.

**FIGURE 3:**
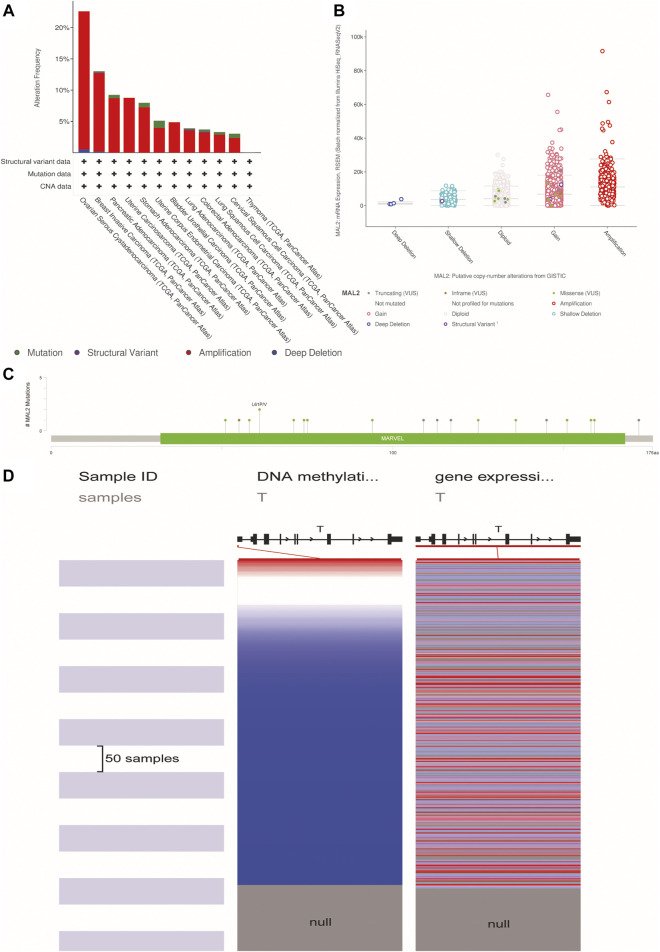
The alteration of MAL2 in pan-cancers. **(A)** MAL2 mutation level in The Cancer Genome Atlas (TCGA) database. **(B)** Putative copy-number alterations from GISTIC. **(C)** Mutation diagram of MAL2 across protein domains. **(D)** Heatmap of MAL2 DNA methylation and mRNA expression by the Xena browser.

To further explore the MAL2 mutation in pan-cancer, we mined data from COSMIC and cBioPortal, which provide information about different kinds of mutation, including non-sense mutation, missense mutation, and synonymous mutations. As showed in Figure 2, missense mutations were obvious in breast tissues (2.17%), large intestine (18.52%), lung tissues (50%), ovarian tissues (13.99%), pancreatic tissues (2.7%), stomach tissues (50%), and uterine endometrial tissues (30%), while synergistic mutations and non-sense mutations were rare and only found in few samples out of total samples. The mutation samples are mostly in OC, and the most common mutations in MAL2 coding chain are C > T (41.25%) and G > A (31.25). Figure 3A–C shows data from cBioPortal based on TCGA database, indicating that there were 18 mutation sites in MAL2, located between amino acids 0 and 176 (Figure 3C). In general, the mutation rate was higher in ovarian serous cystadenocarcinoma and invasive breast carcinoma (Figure 3A). Copy number alteration is another kind of mutation, and the most common copy number alterations are gain and amplification (Figure 3B). The UCSC Xena online tool is used to analyze the relationship between gene expression and DNA methylation. We explored the DNA methylation of the promoter region of MAL2 in OC sample. Data revealed extensive MAL2 gene hypomethylation in OC tissue samples, which is consistent with the high expression of MAL2 in OC, indicating that the promoter hypomethylation of MAL2 may be the mechanism of the high expression of MAL2 (Figure 3D). These outcomes may help to further understand the functional mechanism of MAL2 in OC.

### Functional Enrichment and PPI Network of MAL2 in Patients With OC

**FIGURE 4:**
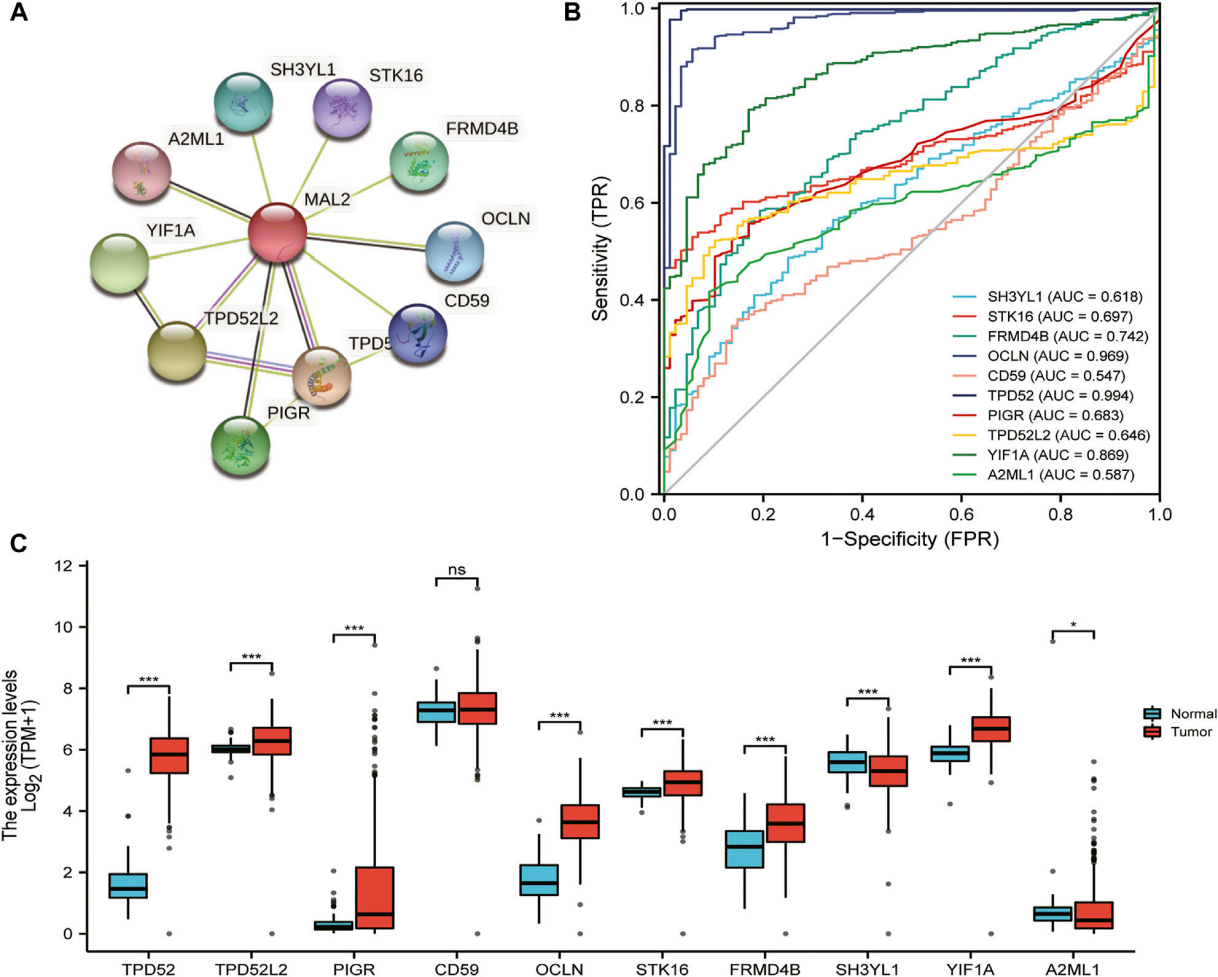
Functional enrichment and protein–protein interaction (PPI) network of MAL2 in patients with OC. **(A)** PPI network by STRING database. **(B)** Areas under the curve (AUCs): 10 hub genes’ AUCs in predicting prognosis of OC patients. **(C)** The expression analysis of 10 hub genes in OC.

Then, we explored the function of MAL2 and their protein interactions by constructing a PPI network of MAL2 by STRING database. The PPI network diagram contains MAL2 and 10 related proteins (Figure 4A). The latter include TPD52, TPD52L2, PIGR, CD59, OCLN, FRMD4B, STK16, SH3YL1, A2ML1, and YIF1A (Figure 4A). We further investigated the expression of the 10 hub genes based on the data from GTEx (*n* = 88) and TCGA (*n* = 427). As listed in Figure 4C, TPD52, TPD52L2, PIGR, OCLN, FRMD4B, STK16, A2ML1, and YIF1A were significantly higher in OC cancer tissues (*p* < 0.05), while SH3YL1 was significantly lower in OC (*p* < 0.05), and CD59 showed no statistical expression in OC tissues compared with normal tissues (*p* > 0.05). Finally, in order to evaluate the specificity of the 10 hub genes for detecting OC patients, we achieved mRNA expression data to construct a ROC curve. Among these curves, hub genes TPD52 (AUC = 0.994), OCLN (AUC = 0.969), and YIF1A (AUC = 0.869) achieved the highest AUC score in detecting OC, followed by FRMD4B with AUC 0.742, STK16 with AUC 0.697, and PIGR with AUC 0.683, whereas the detection accuracy of other hub genes (TPD52L2, SH3YL1, A2ML1, and CD59) ranged from 0.547 to 0.646 (Figure 4B).

### GGI Network and Drug Sensitivity of MAL2 in OC

**FIGURE 5:**
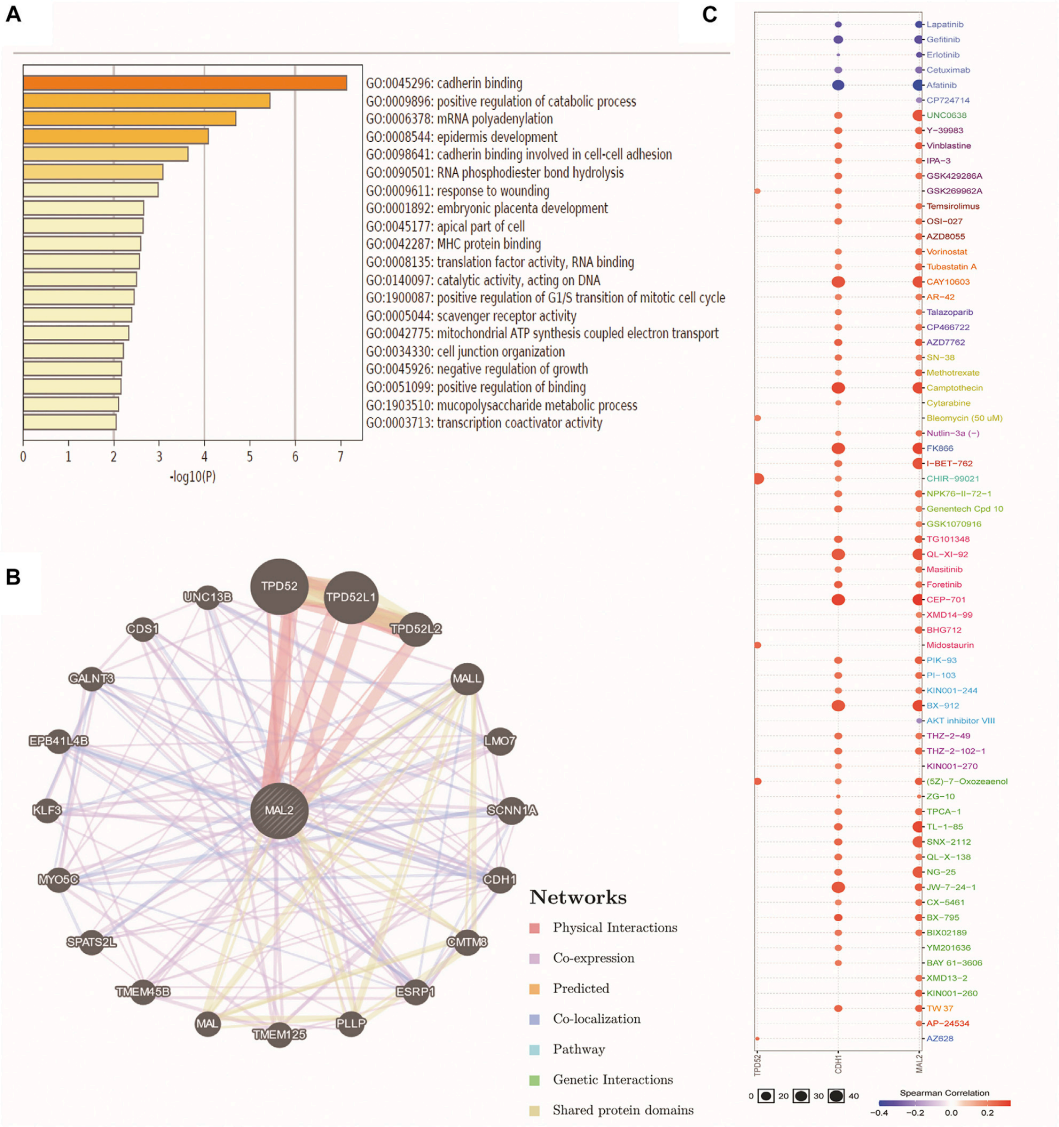
Gene–gene interactionGGI) and drug sensitivity of TPD52, CDH1, and MAL2 in OC. **(A)** The analysis of GO by SangerBox online platform. **(B)** The network for MAL2 and the 20 most frequently altered neighbor genes by GeneMANIA. **(C)** Drug sensitivity analysis of TPD52, CDH1, and MAL2 in OC patients by the GSCALite online tool. The positive correlation means that a high expression of TPD52, CDH1, and MAL2 is resistant to the drugs.

We further explored the functional mechanism of MAL2 in OC from the perspective of bioinformatics. The function of MAL2 and significantly associated genes was predicted by analyzing GO from the Metascape database. GO enrichment analysis revealed that MAL2 mainly participated in cadherin binding, cadherin binding involved in cell–cell adhesion, epidermis development, and so on (Figure 5A). Subsequent exploration was performed to analyze the potential role of MAL2 in drug sensitivity of OC using the GSCALite online tool. GeneMANIA was used to construct the GGI network for MAL2 and the 20 most altered associated genes. The data showed that MAL2 was strongly correlated to TPD52, which has been reported to be a molecular chaperone of MAL2 (16). Among these genes, CDH1 is closely associated with MAL2 alterations, which is consistent with our results above (Figure 5B). In order to further understand the clinical significance of MAL2, we selected the top three hub genes (MAL2, CDH1, and TPD52) from the GGI network to analyze their potential roles in drug susceptibility in OC according to the Cancer Therapeutics Response Portal (CTRP). The result showed that the high expression of CDH1 was resistant to 51 drugs or small molecules, and the high expression of MAL2 was also resistant to 52 drugs or small molecules, indicating that MAL2 have the potential ability of mediating drug sensitivity in OC (Figure 5C).

### MAL2 Expression in OC Cell Lines

**FIGURE 6:**
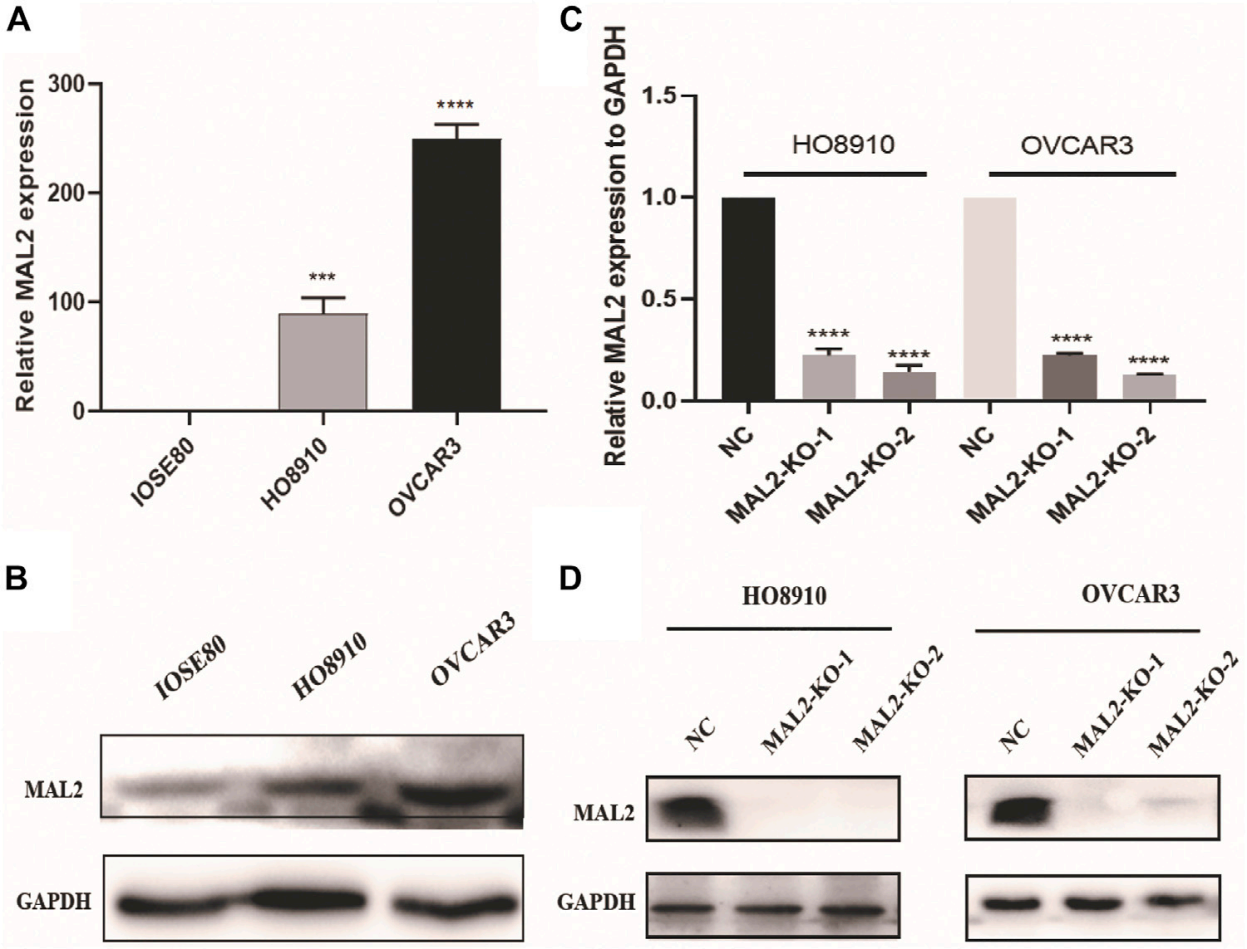
The expression of MAL2 in OC cell lines. **(A)** and **(C)** The expression status of MAL2 was analyzed by real-time quantitative polymerase chain reaction (qRT-PCR). *p < 0.05; **p < 0.01. **(B)** and **(D)** The relative expression of MAL2 (compared with that of GAPDH) was examined *via* western blotting.

To verify the results obtained by data mining, the expression of MAL2 was detected in the OC cell lines HO8910 and OVCAR3 and the human ovarian epithelial immortalized cell line IOSE80 using real-time quantitative polymerase chain reaction (qRT-PCR) and western blotting. As shown in Figure 6A, B, OVCAR3 and HO8910 cells had higher expression levels of MAL2 than the normal ovarian epithelial cell line IOSE80; therefore, OVCAR3 and HO8910 were selected for subsequent experiments. To investigate the potential role of MAL2 in OC, we aimed to knock out MAL2 using the CRISPR/Cas9 system. We designed two MAL2-targeting sgRNAs directly targeting the exon 1 of MAL2. The recombinant vector was packaged into lentivirus, which was then used to infect OC cells. To identify the knockdown efficiency, qRT-PCR was used to determine the mRNA expression level of MAL2 (Figure 6C). Then, western blotting confirmed that the protein expression of MAL2 in those monoclonal cell lines was knocked out (Figure 6D). In the next experiment, we mainly show the experimental results of the KO-2 group. For the convenience of writing, we use KO to represent KO-2.

### Knockout of MAL2 Inhibits the Proliferation and Promote Apoptosis of OC Cells *In Vivo* and *In Vitro*

**FIGURE 7:**
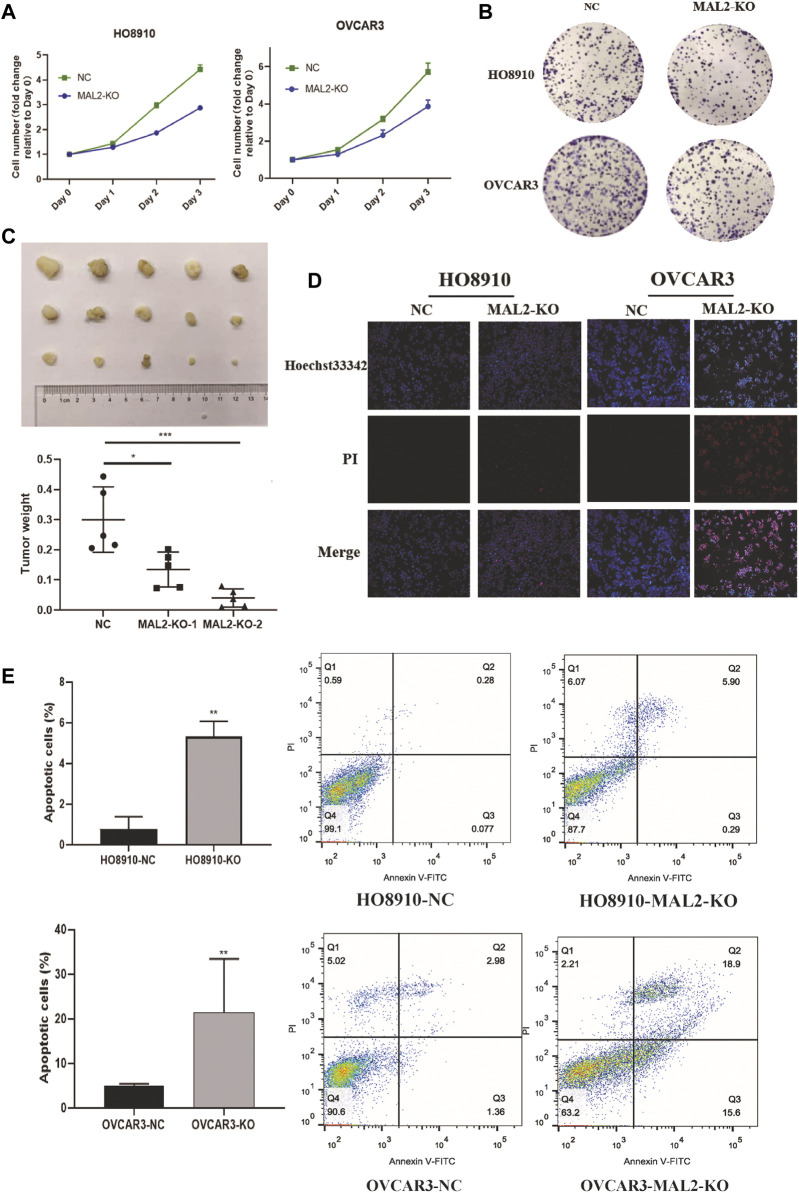
Knockout of MAL2 in OC cells inhibits proliferation and apoptosis. **(A)** HO8910 and OVCAR3 cell lines transfected with sgRNA or negative control (NC) were cultured in 96-well plates for 3 days, with 1,000 cells per well, and Cell Counting Kit-8 (CCK-8) assays were used to measure cell proliferation. **(B)** HO8910 and OVCAR3 cell lines transfected with sgRNA or NC were cultured in six-well plates for 10–14 days, with 1,000 cells per well. **(C)** Nude mice were injected with MAL2 knockout cells and NC cells, with 5 × 10^6^ cells per mice. The weights of the tumors of each group were recorded. **(D)** Representative photomicrographs of HO8910 and OVCAR3 cells stained with Hoechst 33342 and propidium iodide (PI). Necrotic cells exhibited strong red and weak blue staining, appearing purple in the merged image; apoptotic cells exhibited strong blue and weak red staining with condensed or fragmented nuclei, and normal viable cells exhibited weak blue and weak red staining. **(E)** The apoptotic rate was analysis by flow cytometry.

As proliferation and apoptosis are two key hallmarks of cancer, we performed CCK-8 and colony formation assays to evaluate the effect of MAL2 expression on cellular proliferation. The absorbance values and colony numbers of MAL2 knockout cells were notably decreased compared with those of negative control (NC) cells (Figure 7A, B). In xenograft mice, knockout of MAL2 dramatically alleviated tumor growth (Figure 7C). The results of the *in vivo* experiment further confirmed that the knockout of MAL2 efficiently suppressed the proliferation of OC cells. To study whether the knockout of MAL2 could increase apoptosis rate in ovarian cancer cells, cell apoptosis was visualized by fluorescence microscopy, and the results revealed that MAL2 suppressed apoptosis (Figure 7D). We further performed the flow cytometric analysis of annexin V and PI double staining to detect the apoptotic rate in ovarian cancer. The total apoptotic cells increased significantly in the MAL2-knockout groups (Figure 7E). These results suggest that MAL2 can promote OC cell proliferation and apoptosis.

### Knockout of MAL2 Inhibits the Migration and Invasion of OC Cells

**FIGURE 8:**
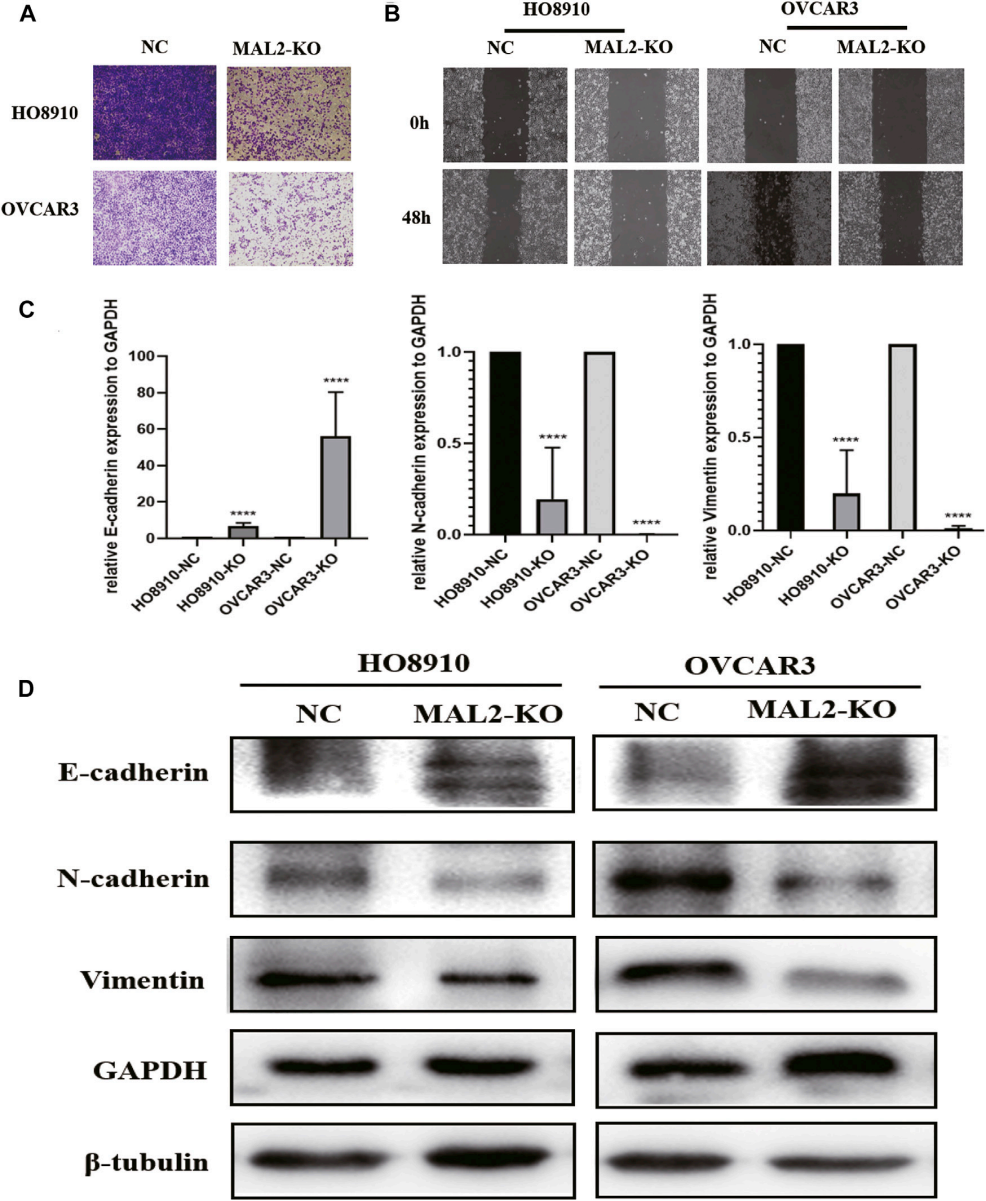
Knockout of MAL2 in OC cells inhibits migration, invasion, and epithelial-mesenchymal transition (EMT). **(A)** Transwell migration assays in MAL2 knockout HO8910 and OVCAR3 cells and their corresponding negative control cells, with 8 × 10^4^ cells per well. **(B)** Wound healing assays in MAL2 knockout HO8910 and OVCAR3 cells and their corresponding negative control cells. **(C)** qRT-PCR analysis of the expression levels of E-cadherin, vimentin, and N-cadherin in HO8910 and OVCAR3 (KO or NC) cell lines. *p < 0.05; **p < 0.01. **(D)** Western blotting analysis of the expression levels of E-cadherin, vimentin, and N-cadherin in HO8910 and OVCAR3 (KO or NC) cell lines.

Metastasis is a crucial characteristic of OC and causes poor outcomes in patients. To investigate the effect of MAL2 on the cellular migration and invasion of OC cells, Transwell experiments and wound healing assays were performed *in vitro*. As shown in Figure 8A, B, the downregulation of MAL2 inhibited the migration and invasion of OC cells. EMT plays an essential role in transforming epithelial cells into mesenchymal cells with a more aggressive phenotype, which is associated with tumor cell invasion and metastasis. EMT-related protein markers are dysregulated in this process; for example, N-cadherin and vimentin are upregulated and E-cadherin is downregulated. Interestingly, we observed that MAL2 knockout had an increased expression of E-cadherin and a decreased expression of N-cadherin and vimentin (Figure 8C, D). Collectively, these results suggested that knockout of MAL2 inhibits EMT in OC, which might be a potential mechanism of cell metastasis and invasion in OC.

## Discussion

In this study, we profiled a comprehensive investigation of the characteristics of MAL2 across 13 types of cancer based on multiple databases, including expression, survival analysis, and gene mutation. The data showed that MAL2 was increased in most types of tumors and is obviously associated with prognosis of patients with BLCA, BRCA, OC, PAAD, STAD, THYM, and UCEC. Thus, MAL2 may serve as a promising biomarker for diagnosis and prognosis for BLCA, BRCA, OC, PAAD, STAD, THYM, and UCEC. The results of cBioPortal, COSMIC, and UCSC Xena databases revealed that changes in the MAL2 gene mainly occurred in BRCA and OC. The mutation rate of non-sense substitution and synonymous substitution was the highest in cervical tissues (both 33.33%), missense substitution (50%) in lung tissues, while OC has the most mutation types with the largest sample accounts, indicating that MAL2 have a potential role in OC. Therefore, further study needs to be performed to uncover the mechanism of MAL2 gene alteration. Based on the bioinformatics analysis above, the mechanism of this abnormal expression may be related to the hypomethylation of the MAL2 gene promoter region, which is consistent with the high expression of MAL2 in OC (Figure 3). GO analysis indicated that MAL2 contribute to cadherin binding and cadherin binding involved in cell–cell adhesion of OC. Therefore, one of the possible mechanisms of MAL2 in cancer may be related to the relevant signal pathway regulating cell adhesion.

PPI and GGI networks revealed MAL2-related proteins and genes, which also play an important role in detecting OC patient outcome and affecting drug sensitivity. ggplot2 package and ROC package of R3.6.3 version were used to explore the expression and detection value of MAL2-related proteins in OC. It seemed that OCLN and TPD52 may be the most promising biomarker for detecting OC patients. At present, some literatures have reported that TPD52 is highly expressed in ovarian cancer tissues (Byrne et al., 2005) and acts as a downstream signal molecule in some processes (Liu and Xi, 2020), indicating that TPD52 also plays an important role in the occurrence and development of ovarian cancer, and MAL2 and TPD52 are chaperones. They are likely to play a synergistic role in the occurrence and development of ovarian cancer. Later, we verified the database analysis results at the cellular level and found that the expression of the MAL2 was upregulated in the OC cell lines HO8910 and OVCAR3 compared with a normal ovarian epithelial cell line. Functional experiments revealed that MAL2 can promote cell proliferation, invasion, and metastasis and regulation of EMT in OC cell lines. Collectively, our results indicated that MAL2 may affect the malignant progression of OC cells. The analysis results showed that MAL2 is remarkably overexpressed in OC, which is correlated to poor clinical outcome in Kaplan–Meier survival curve analysis.

OC is the most lethal disease in the female reproductive system with the most histopathological types. Hundreds and thousands of researches have reported various genes related to OC proliferation, invasion, and metastasis. However, specific biomarkers for diagnosis, therapy, or prognosis are still insufficient. Therefore, it is urgent to explore novel biomarkers for OC. MAL2 has been reported to be highly expressed in several types of epithelial tumors, such as cervical cancer (Obermayr et al., 2010), and is related to cell invasion and metastasis (Li et al., 2017). Fischer et al. (2015) found that MAL2 was upregulated in blood samples taken from OC patients, which is consistent with the expression data from databases. The results showed that MAL2 mRNA level was upregulated in multiple types of cancer, especially in OC, and MAL2 expression is significantly correlated with clinical stage among cancers. Drug sensitivity analysis suggested that MAL2 may be a potential drug target for OC chemotherapy. To verify the bioinformatics predictions regarding the MAL2 in clinical detection and therapeutic value, we further performed experiments *in vivo* and *in vitro* and found that MAL2 downregulation effectively inhibited xenograft growth in nude mice and suppressed proliferation in OC cells.

Cell polarity changing is one of the important differences between epithelial cells and mesenchymal cells, which is a complicated biological process. Previous studies have found that OC cells have a wide range of EMT status (Tan et al., 2013), and this phenotype is related to the overall survival rate and disease-free survival rate of patients (Tan et al., 2014). The functional enrichment analysis showed that MAL2 is involved in cadherin binding and cell adhesion. A previous study revealed that MAL2 is located on the surface of the cell membrane and can maintain cell polarity. Tumor invasion and metastasis are multifactor and multistep processes that are regulated by a variety of genes. EMT has been reported to be involved in this process, which is associated with a poor prognosis. EMT is associated with aberrant expression levels of related cytokines and transcription factors; for example, the epithelial phenotype marker E-cadherin is downregulated and the mesenchymal phenotype markers vimentin and N-cadherin are upregulated during EMT (Fischer et al., 2015). As an epithelial biomarker, E-cadherin has been proven to play an important role in tumor metastasis (Rötzer et al., 2015; Shen and Sun, 2018; Wu et al., 2018). Studies have found that CHIP functions as an oncogene by inhibiting E-cadherin (Xu et al., 2018). MiR-30a can inhibit EMT and metastasis of triple-negative breast cancer cells by targeting ROR1 (Wang et al., 2018). In our study, western blotting was used to detect the expression of EMT-related proteins at the cellular level. Downregulation of MAL2 increased the expression of E-cadherin at the protein level but decreased the expression of vimentin and N-cadherin, suggesting that MAL2 may affect the proliferation, migration, and invasion of OC cells by regulating EMT. Considerable evidence has declared that a variety of signaling pathways, such as the Wnt/β-catenin pathway and PI3K/Akt pathway, are involved in the regulation of EMT. EMT was considered to be related to the proliferation, apoptosis, invasion, and migration of OC cells (Liang et al., 2018; Mitra et al., 2017; Zhang et al., 2018). Therefore, exploring the upstream targets regulating EMT in OC is of great significance for finding new molecular therapeutic targets.

In conclusion, we profiled MAL2 expression, survival rate, and mutation data across pan-cancers, which revealed the potential role of MAL2 in multiple types of cancer. Then, we focused on the data mining of MAL2 methylation, function enrichment, PPI and GGI networks, cancer pathway, and drug sensitivity in OC, indicating that MAL2 may be a potential drug target of OC. To further verify the data from databases, we obtained important phenotypic results and demonstrated that the downregulated expression of MAL2 is associated with the proliferation, migration, and EMT of OC cells. However, there are still some limitations in our study. First, further clinical work is required to provide more convincing results. Second, we need to conduct more *in vitro* experiments to better verify the mechanism of MAL2. In addition, the downstream proteins of MAL2 need to be assessed in further studies. As MAL2 is a membrane protein located on the surface of the cell membrane, previous studies have confirmed that it binds to TPD52. In this study, TPD52 was also assessed, but further research should be carried out to verify the expression of TPD52 and its interaction with MAL2 in OC.

## Data Availability

The original contributions presented in the study are included in the article/Supplementary Material; further inquiries can be directed to the corresponding authors.
